# Impact of Climate Change on Farm Animals: Physiological Mechanisms, Health and Productivity, and Integrated Adaptation Strategies

**DOI:** 10.3390/vetsci13090954

**Published:** 2026-09-12

**Authors:** Mahmoud Kamal, Yasser Alrauji, Mahmoud Roshdy, Hassan A. Khalil, Mostafa A. Ayoub, Mohamed Shehab-El-Deen

**Affiliations:** 1 Animal Production Research Institute, Agricultural Research Center, Dokki, Giza 12618, Egypt; m_greash@yahoo.com; 2 Department of Animal and Poultry Production, College of Agriculture and Food, Qassim University, Buraydah 52571, Saudi Arabia; 3 Animal Production Department, Faculty of Agriculture, Suez Canal University, Ismailia 41522, Egypt; mahmoud_roshdy@agr.suez.edu.eg (M.R.); hkhalil011971@agr.suez.edu.eg (H.A.K.); mostafa_ayoub@agr.suez.edu.eg (M.A.A.)

**Keywords:** hyperthermia, thermoregulation, climate resilience, livestock productivity, oxidative stress

## Abstract

Climate change is accelerating the frequency of extreme thermal environments, threatening animal welfare, global herd productivity, and agricultural economics. Heat stress—occurring when the ambient temperature-humidity index (THI) surpasses species-specific thermoneutral thresholds—initiates physiological heat-loss cascades (panting, sweating, and peripheral vasodilation), neuroendocrine disruptions (depressed thyroid hormones, elevated glucocorticoids, and insulin dysregulation), mitochondrial oxidative stress, and gut-barrier breakdown. These systemic disruptions drive substantial performance declines: voluntary dry matter intake drops by 15–25%, average daily gain by 15–30%, milk yield by 10–25%, conception rates by 30–50%, and poultry egg output and shell integrity by 15–25%. Species-specific vulnerability is governed by intrinsic anatomical and metabolic differences, including the substantial fermentation heat increment in ruminants, the lack of functional sweat glands and heavy feather insulation in poultry, and the low cutaneous evaporative capacity of swine. Furthermore, thermal stress induces oxidative damage via free radical accumulation and alters blood composition, triggering severe immunosuppression that heightens susceptibility to mastitis, respiratory complexes, and metabolic endotoxemia. Mitigation requires an integrated solutions matrix combining environmental cooling (tunnel ventilation, pad cooling, and shade), targeted nutrition (antioxidants, organic trace minerals, osmolytes, and rumen-protected fats), genomic selection (SLICK locus and thermotolerant crossbreeding), and real-time Precision Livestock Farming (PLF) monitoring. Addressing these challenges is paramount to ensuring sustainable livestock production, animal welfare, and global food security under impending climate scenarios.

## Simple Summary

This review provides an integrated synthesis of the impacts of climate change-induced heat stress on the physiological, metabolic, and cellular mechanisms of major livestock species (ruminants, monogastrics, and poultry), alongside advanced mitigation strategies. Economic losses are profound, exceeding an estimated $59–86 billion globally per year, driven by heat-induced declines in voluntary feed intake, daily weight gain, milk production, reproductive conception rates, and egg yield. With climate models projecting an increased frequency and severity of extreme thermal events, achieving sustainable livestock resilience necessitates coordinated interventions spanning molecular nutrition, environmental engineering, genomic selection, and Precision Livestock Farming (PLF).

## 1. Introduction

Anthropogenic global warming constitutes an urgent environmental threat that profoundly destabilizes livestock biology and farm economics, thereby challenging global food security [1,2]. High-resolution modeling of current climate projections predicts that extreme heat events are increasing in frequency, duration, and intensity, fundamentally changing the thermal environment experienced by livestock globally [3]. Animals have a narrow thermal range for normal metabolism; hence, the livestock sector is very vulnerable, and climate change threatens the adaptive capacity of many production systems [4]. Without effective mitigation and adaptation strategies, some areas are projected to become unsuitable for traditional outdoor livestock production later this century [5].

Heat stress is a physiological state in which the challenge of the environment exceeds the ability of the animal to dissipate heat and achieve thermal equilibrium. Most commonly, this is caused by the temperature-humidity index [6]. The Shared Socioeconomic Pathway (SSP5-8.5) projects a twelvefold increase in annual hours exceeding severe heat-stress thresholds (THI ≥ 84, representing a critical emergency boundary for cattle, pigs, and poultry) by the late 21st century [7]. Increased temperatures, humidity, and changes in precipitation pose complex challenges to livestock production in relation to feed supply, water availability, and disease dynamics [8]. Animals have several compensatory mechanisms to cope with thermal stress, and these include increased respiration, perspiration in those species with functional sweat glands, and peripheral tissue vasodilation. However, these responses are energy-exhausting and often not enough in prolonged exposure [9]. The first signs of performance deficits are therefore in feed intake and energy balance, followed by growth, lactation, reproduction, immunity, and overall survival [10].

One of the high costs of climate change, and one that is underappreciated, is the heat stress on the world’s livestock industry. Annual losses are a threat to the economic sustainability of livestock farms in different production systems and geographical areas. These losses are caused by direct production losses, increased veterinary costs, decreased reproductive efficiency, and mortality costs [5,11]. These economic pressures are not only in intensive farming systems, but also in extensive systems, which are challenged by decreasing climatic suitability and forage quality with increasing heat and aridity. Heat stress is a multifactorial problem affecting animal health, farm design, feed efficiency, environmental sustainability, and climate resilience [12]. Lactating dairy cows under heat stress account for approximately $1.0 to $1.8 billion of the annual losses of the US national dairy industry, or 64% of total losses. These include decreased milk production during heat waves (10–25%) in 2024 [13], reduced milk quality, increased culling, and lower conception rates. Dry dairy cows also experience heat stress effects, with an additional annual revenue loss of $810 million due to decreased reproductive performance and increased mortality [14].

Beyond the dairy sector, thermal stress exerts comparable devastation across other livestock sectors. In the swine industry, seasonal hyperthermia causes an estimated annual loss exceeding $300 million to $1 billion in the United States alone, primarily driven by reduced average daily gain, impaired feed conversion efficiency, prolonged days to slaughter, and compromised sow fecundity (seasonal infertility and smaller litter sizes) [15]. Similarly, the global poultry sector suffers billions of dollars in losses annually; commercial broilers show severe declines in growth rates and increased heat-induced mortality, while laying hens and breeders experience sharp reductions in egg production, reduced eggshell thickness, and lower fertility [16]. Small ruminants are likewise vulnerable; under prolonged hyperthermia, heat-stressed sheep and goats suffer notable drops in reproductive performance (poor oocyte and semen quality, early embryonic demise) as well as substantial reductions in meat yield and daily milk synthesis, particularly among high-yielding exotic breeds unadapted to semi-arid agro-climatic zones [17]. Integrating these multifaceted multi-species losses underscores the pervasive threat of climate change to global livestock production.

This review focuses on recent projections of climate change and the physiological and production response of livestock to thermal stress, as well as mitigation/adaptation strategies (behavioral, nutritional, genetic, and management). It identifies knowledge gaps and gives evidence-based recommendations for researchers, veterinarians, and policymakers to improve thermal resilience and sustain productivity.

### Literature Search Methodology

To ensure scientific rigor and transparent literature mapping, a comprehensive electronic search was conducted across Web of Science, Scopus, PubMed/MEDLINE, and Google Scholar databases covering peer-reviewed articles published from January 2000 through February 2026. Search strings combined Boolean operators with specific MeSH terms: (‘heat stress’ OR ‘thermal stress’ OR ‘hyperthermia’) AND (‘livestock’ OR ‘dairy cattle’ OR ‘poultry’ OR ‘swine’ OR ‘sheep’ OR ‘goats’ OR ‘rabbits’) AND (‘physiological mechanisms’ OR ‘gut barrier’ OR ‘oxidative stress’ OR ‘immune function’ OR ‘epigenetics’ OR ‘microbiota’ OR ‘mitigation’ OR ‘precision livestock farming’). Inclusion criteria encompassed original empirical studies, validated meta-analyses, and peer-reviewed reviews reporting quantifiable physiological, endocrine, cellular, or productive responses to elevated THI. Non-peer-reviewed conference abstracts, non-English publications without verified translations, and studies lacking defined environmental parameters were excluded. A total of 116 core reference papers were systematically selected, synthesized, and contextualized.

## 2. Physiological Mechanisms of Heat Stress

The primary physiological responses used during heat stress are coordinated to preserve core temperature. These start with behavioral countermeasures such as reduced activity, seeking shade, and water intake, followed by evaporative cooling and re-routing blood flow to the cardiovascular system [18]. When the ambient heat load exceeds the animal’s ability to dissipate heat, panting, sweating (in species with functional sweat glands), and peripheral vasodilation are the principal thermoregulatory mechanisms, but these responses are associated with substantial energetic and metabolic costs that reduce performance and health [19]. Panting increases evaporative water loss from the respiratory tract but is less effective at high humidity. Sweating is an important cooling route in ruminants and horses but minimal in swine and poultry, explaining important species differences in heat tolerance [20]. Peripheral vasodilation markedly increases cutaneous blood flow to facilitate convective and radiative heat dissipation. However, this physiological trade-off triggers compensatory splanchnic vasoconstriction, reducing intestinal and mesenteric blood perfusion by up to 40–50%. This splanchnic hypoperfusion starves the intestinal mucosa of oxygen and energy, causing enterocyte hypoxia, oxidative injury, and tight-junction degradation [21]. Concurrently, the observed insulin resistance during thermal challenge is governed by a dual, synergistic pathway: first, extended elevation of circulating glucocorticoids (cortisol) promotes gluconeogenesis while directly antagonizing peripheral GLUT4-mediated glucose uptake; second, systemic translocation of luminal lipopolysaccharides (LPS) across the compromised gut barrier stimulates TLR4/JNK cascades, causing inhibitory serine phosphorylation of Insulin Receptor Substrate-1 (IRS-1) and impairing downstream insulin signaling [9].

**Figure 1:**
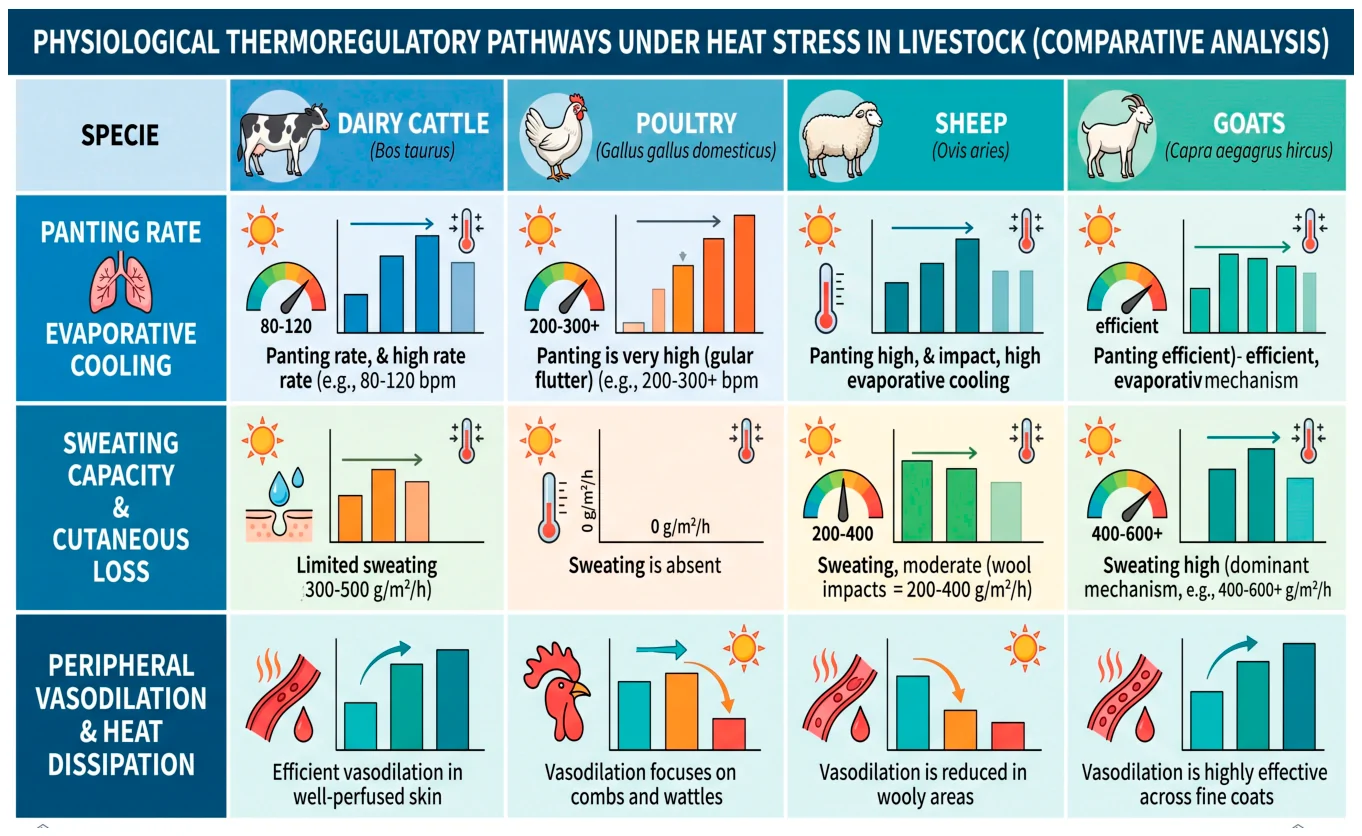
Comparative schematic of species-specific thermoregulatory mechanisms under heat stress in dairy cattle, poultry, sheep, and goats. In the figure, horizontal axes indicate rising heat stress progression, while vertical axes reflect response intensity. Upward blue arrows signify effective, sustained cooling pathways, whereas downward orange arrows denote declining efficacy. Dial gauges illustrate reliance levels on specific mechanisms, scaling from low (green) to maximal physiological engagement (red).

Moreover, heat stress also induces important endocrine and metabolic adaptations that lead the animal to switch from a production to a survival mode. Heat stress generally reduces thyroid hormone levels, resulting in a lower basal metabolic rate and endogenous heat production but also lower growth, milk synthesis, and other productive processes [22]. At the same time, cortisol is up-regulated as part of the stress response to help mobilize energy, but extended elevation causes catabolism, insulin resistance, and immune dysfunction [9]. These hormonal changes change the partitioning of nutrients, reduce glucose use by productive tissues, increase the use of body reserves, and lead to negative energy balance, impaired growth, reduced milk yield, and poorer reproductive performance [23]. Figure 1 illustrates the distinct, species-specific physiological adaptations across dairy cattle, poultry, sheep, and goats to achieve thermal equilibrium under elevated ambient temperatures. Marked disparities exist in evaporative and non-evaporative heat dissipation pathways among these livestock species:

Dairy Cattle: As major metabolic heat producers, lactating cows rely heavily on peripheral vasodilation and substantial cutaneous sweating as their primary defense against thermal load. However, as ambient humidity approaches saturation, sweating efficiency declines, triggering intense compensatory panting (tachypnea) to facilitate respiratory evaporative cooling, which imposes severe energetic trade-offs.

Poultry: Domestic fowl lack functional sweat glands because of their integumentary structure and feather coverage. Consequently, their sweating capacity is negligible, forcing them to rely heavily on panting (gular fluttering) as their paramount route for evaporative cooling (~60–70% of total dissipation during peak heat). Peripheral vasodilation is largely restricted to non-feathered appendages (comb, wattles, and shanks).

Small Ruminants (Sheep and Goats): Both species demonstrate an intermediate adaptive profile characterized by behavioral thermoregulation and efficient panting. Sheep possess limited sweating capacity due to the insulative fleece barrier, depending predominantly on rapid shallow panting. Conversely, goats exhibit higher sweating rates per unit surface area, greater resilience in cutaneous water loss, and superior peripheral vasomotor responses compared to wool sheep, conferring enhanced thermotolerance in arid climates.

The matrix illustrates the relative functional reliance and physiological capacity across three cardinal heat-dissipation pathways: (A) Panting Rate and Respiratory Evaporation, (B) Cutaneous Sweating Capacity, and (C) Peripheral Vasodilation and Sensible Heat Loss. Relative capacities and responsiveness are normalized across species to highlight key evolutionary and morphological differences (such as the absence of cutaneous sweating in avian species vs. active cutaneous sweating in dairy cattle and distinct fleece/feather insulation constraints) [16,24,25].

Heat stress induces oxidative stress at the cellular level through stimulation of reactive oxygen species (ROS) production and impairment of antioxidant defenses. High temperature leads to a breakdown of electron transport and increased leakage of electrons that form superoxide and downstream oxidants, making mitochondria a major source of this ROS generation [26]. The major antioxidant systems, such as superoxide dismutase, catalase, and glutathione peroxidase, may be overwhelmed, and lipid, protein, and DNA damage increases due to the depletion of non-enzymatic antioxidants [27]. Heat shock proteins are induced to protect the cells by stabilizing the proteins, reducing aggregation and promoting refolding, especially HSP70. They also increase the cells’ resistance to apoptosis and inflammation [28]. However, the synthesis of HSPs is itself energetically expensive, and so this defense can compete with productive metabolism [29].

Heat-induced mucosal hypoxia and oxidative stress fundamentally reshape the luminal microenvironment, driving severe gastrointestinal dysbiosis [30]. In both ruminants and monogastrics, populations of beneficial obligate anaerobes (including *Fibrobacter succinogenes*, *Ruminococcus albus*, and butyrate-producing *Faecalibacterium prausnitzii*) are drastically depressed, resulting in a 30–45% decline in total short-chain fatty acid (SCFA; acetate, propionate, and butyrate) production [31,32]. Conversely, opportunistic facultative pathogens such as *Escherichia coli*, *Salmonella enterica*, and *Clostridium perfringens* proliferate. The depletion of luminal butyrate directly compromises enterocyte mitochondrial ATP production and impairs tight-junction assembly (claudin-1, occludin, and ZO-1), facilitating the systemic translocation of endotoxins (LPS). Circulating LPS engages TLR4/NF-κB inflammatory signaling, exacerbating systemic inflammation, muscle proteolysis, and nutrient partitioning deficits [31].

These physiological, endocrine, oxidative, and gastrointestinal changes are working in unison to reduce productivity, impair welfare, and increase disease susceptibility [21]. The combination of these effects explains heat stress’s impact on reduced feed intake, growth, reproductive efficiency, milk production, and carcass quality, as well as an increased requirement for antioxidant, nutritional, and management interventions [33]. Therefore, strategies such as cooling, shade, ventilation, electrolyte support, antioxidants, probiotics, and gut-protective nutrients are central to alleviating heat stress in livestock [34].

## 3. Impacts on Animal Performance

**Figure 2:**
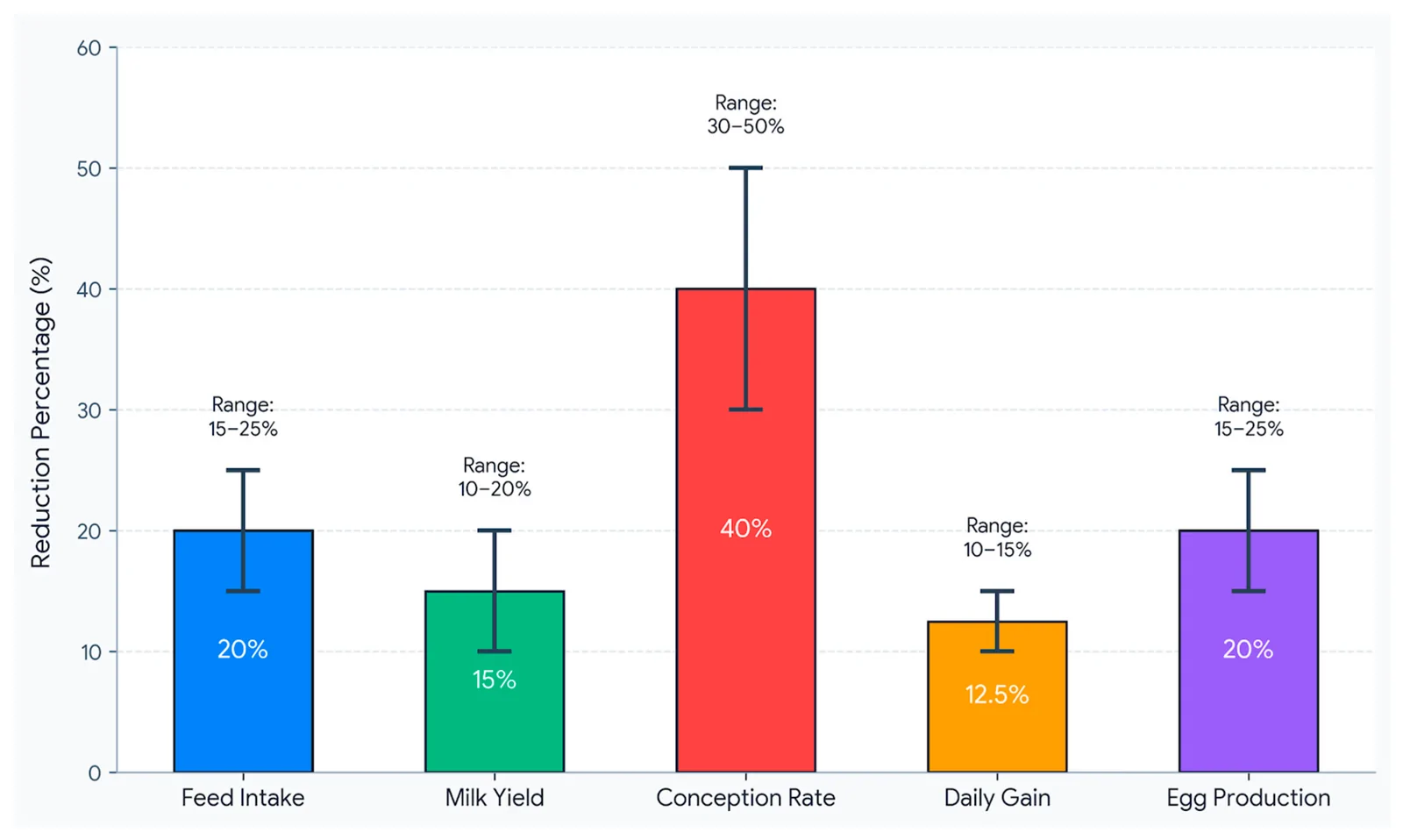
Performance reduction due to heat stress across livestock production traits.

Heat stress causes reductions in feed intake, growth, milk production, reproductive efficiency and egg production in all livestock species. These effects are related to reduced voluntary consumption and reduced efficiency of nutrient utilization [35,36]. Generally, animals reduce their dry matter intake to lower metabolic heat production, particularly under moderate to severe heat loads. They also switch their feeding pattern to cooler hours, eat smaller meals, and stand more, a posture adopted to expose more skin to airflow that ultimately suppresses essential rumination cycles and raises maintenance energy costs, further decreasing digestive efficiency. In addition, heat stress reduces the efficiency of nutrient utilization because gut motility is diminished, fiber digestibility is decreased, intestinal absorption is compromised, and metabolic regulation is altered; thus, animals need a higher nutrient density to maintain performance even with reduced intake [6]. Thus, dietary strategies usually aim for higher energy density, targeted amino acid supplementation—using rumen-protected amino acids in ruminants or crystalline and highly digestible sources in monogastrics to lower crude protein levels and attenuate the heat increment of feeding—and electrolyte support to partially compensate for heat-related losses. Figure 2 and Table 1 illustrate the standards of thermal stress performance degradation.

This chart summarizes key performance metric declines across various livestock species as reported in the literature (feed intake: 15–25%; daily gain: 10–15%; milk yield: 10–20%; conception rate: 30–50%; and egg production: 15–25%). Bars represent the mean estimated reduction, with error bars indicating the reported empirical range across diverse production systems and environmental studies [16,24,37,38].

The lower growth performance is attributed to the lower feed intake, the diversion of nutrients to thermoregulation, and the modified endocrine and metabolic responses that reduce protein synthesis and increase catabolism. Poor lean deposition, changes in the muscle-to-fat ratios, and poor meat quality may decrease average daily gain, delay market weight, and affect carcass traits [17]. Heat stress in swine and poultry results in reduced carcass composition and processing value, and in ruminants, reduced marbling and economic value. Critical approaches such as maintenance of energy-dense diets and improvement of environmental cooling can limit these losses in growth [47].

Heat stress is especially common in dairy cows and decreases milk production by decreasing feed intake, altering the partition of nutrients, and directly influencing mammary metabolism. It also influences the composition of milk. It has low fat and protein levels, which reduces the quality of milk. And the payments by processors are lower [48]. These alterations indicate impaired rumen function, oxidative stress, and hormonal imbalances, including decreased insulin-like growth factor signalling and mammary nutrient uptake [22]. The most effective strategies to maintain lactation performance are cooling systems, fresh feed during cooler periods, adequate water access and diet reformulation with bypass fats and protected amino acids [49].

Among the most heat-sensitive production traits is reproductive efficiency, with lower conception rates, reduced oocyte quality, poorer semen quality and increased embryonic loss occurring during hot periods [50]. High body temperature in females affects follicular development, ovulation, progesterone secretion and embryo survival, while in males it affects spermatogenesis, sperm motility and semen viability [51]. These effects may persist after the heat event has passed and reproductive performance may be depressed for weeks. Such losses can be minimized by cooling during breeding, antioxidant supplementation and reproductive technologies such as timed artificial insemination or embryo transfer.

Heat stress in laying hens results in decreased egg production and decreased egg quality, especially shell thickness and shell strength. Reduced feed intake limits the supply of calcium and nutrients, and panting-induced respiratory alkalosis disrupts calcium metabolism and shell formation [43]. These changes lead to more broken eggs, poorer grading quality, and lower market value. Enhanced ventilation, evaporative cooling, calcium and vitamin D supplementation, and electrolyte balance are important measures to maintain laying rate and shell quality under heat stress [44,52].

## 4. Homeostasis and Heat Stress in Poultry

As a representative case for arid and subtropical climates, poultry production systems in environments such as Egypt routinely face sustained ambient temperatures exceeding 30–38 °C for 3–5 continuous months [53,54,55]. Under such thermal loads, blood flow is shunted away from the oviduct toward peripheral thermolytic organs (wattles, comb, and unfeathered skin) [56,57,58,59]. This diversion drastically diminishes the delivery of ionized calcium (Ca^2+^) and bicarbonate to the shell gland (uterus). Concurrently, severe polypnea (panting) expels excess carbon dioxide (CO_2_), inducing acute respiratory alkalosis, lowering blood carbonic acid, and depressing carbonic anhydrase activity in the shell gland lumen, directly causing eggshell thinning and elevated breakage rates [60,61]. Furthermore, chronic heat challenge triggers profound avian hematological shifts: circulating red blood cells (RBC), hemoglobin (Hb), and lymphocytes decline, while circulating heterophils increase significantly, driving a marked elevation in the heterophil/lymphocyte (H/L) ratio, which serves as a primary cellular biomarker of systemic thermal stress [62].

## 5. Species-Specific Vulnerabilities

### 5.1. Ruminant Vulnerabilities

The ruminal fermentation generates considerable metabolic heat, thus adding to the environmental heat load, and this presents a unique thermal problem for ruminants. Fermentation of carbohydrates generates considerable heat—the heat increment of fermentation may account for approximately 20–30% of total metabolic heat production in ruminants, while monogastrics show negligible heat of fermentation. When exposed to heat, animals must dissipate heat from both the environment and from digestion-derived sources, a compounded load that can overwhelm thermoregulatory capacity even at moderate temperatures [39]. High-fiber diets are worse because of the large heat increments produced by fermentation of fiber, leading to a management paradox: fiber is necessary for rumen health, but more fiber means more heat load. This constraint helps explain why ruminants often show heat-stress responses at lower ambient temperatures than predicted by thermoregulatory models and why dietary strategies during hot periods must balance reduced heat increment with maintenance of ruminal function [63].

**Figure 3:**
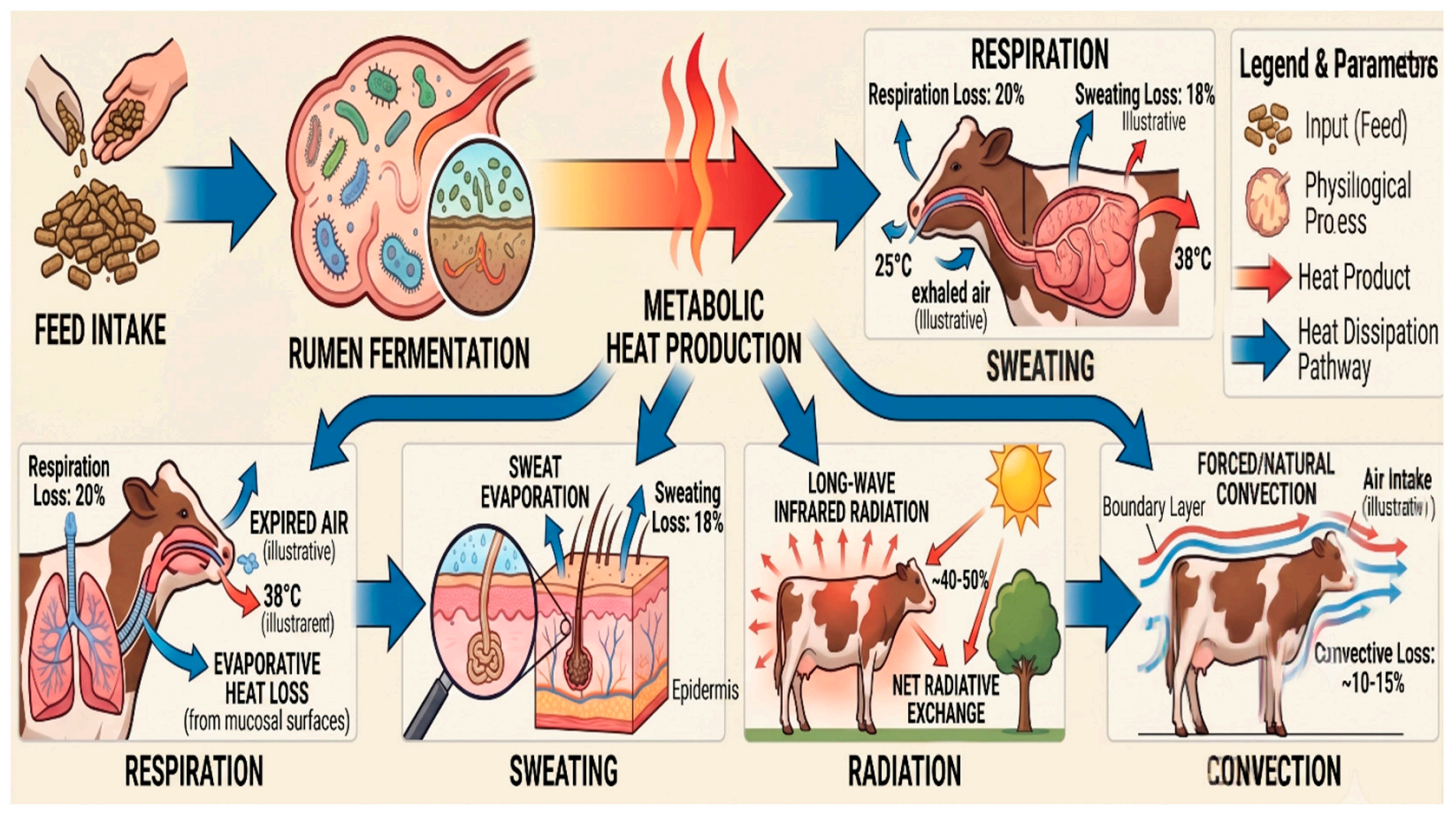
Schematic of heat production and heat loss pathways in the ruminant.

Among ruminants, cattle—especially high-producing dairy breeds like Holstein–Friesian—are most vulnerable. Holsteins show heat-stress responses at relatively low THI values (around THI 68.8 onset; severe effects near THI 84) and can suffer 10–40% milk yield declines, 15–25% reductions in feed intake, and marked reproductive losses (conception-rate declines up to ~30–50%) under heat stress [64,65]. By contrast, Bos indicus breeds (Brahman) display greater thermotolerance via higher sweat rates (2–3× that of Bos taurus), more efficient evaporative cooling, and morphological and genetic adaptations (including the SLICK hair phenotype) that improve heat dissipation and sustain production under thermal stress [40,46]. Breed differences in coat characteristics, skin and sweat-gland physiology, basal metabolic rate, and cardiovascular capacity underline these performance disparities. Sheep and goats occupy intermediate positions: goats are often more heat-tolerant than sheep because of behavioral thermoregulation (shade-seeking, reduced activity), effective water-conservation physiology, and their browsing diets, which can reduce fermentation relative to high-fiber grazing regimes [45]. Figure 3 shows a diagram of heat production and heat loss pathways in ruminants.

This diagram illustrates the cascading flow from energy intake through physiological processes to external dissipation in a ruminant model. Key pathways for metabolic heat generation and environmental heat exchange are clearly defined for physiological analysis.

Mitigation for ruminants must specifically address fermentation-derived heat. Nutritional measures include increasing dietary energy density—with protected (bypass) fats that supply calories without ruminal fermentation—thereby lowering heat increment while supporting energy needs; such strategies have improved milk yield during heat episodes by about 10–15% in some trials [66]. Reducing the amount of dietary neutral detergent fiber (NDF) to safe levels (generally no lower than 28–30% NDF) and selecting fiber sources with a high digestibility will reduce retention time and fermentation heat and will maintain rumen function. Modification of feeding times and frequency (providing fresh rations at cooler times of the day [early morning and evening] and increasing the number of feeding times) helps consumption and reduces the maximum metabolic heat load at high ambient temperatures. Environmental cooling methods such as shade, fans, and sprinklers are important, and comprehensive cooling strategies can reduce milk yield losses by fifty percent and improve average daily gain (ADG) of beef cattle by double-digit percentages during heat events [65]. Long-term strategies involve the genetic introgression of heat-tolerance loci (SLICK) into high-yielding dairy breeds to combine production and resilience [41,67].

### 5.2. Swine Vulnerabilities

Swine are uniquely vulnerable to hyperthermia due to their anatomical lack of functional sweat glands (eccrine glands are restricted to the snout) and a thick insulating layer of subcutaneous adipose tissue [68]. When ambient temperatures exceed their upper critical threshold (22–24 °C for sows and finishing pigs), pigs rely almost exclusively on polypnea (panting) and behavioral thermoregulation (wallowing and floor-contact conduction). Under chronic heat stress, sows experience blunted luteinizing hormone (LH) pulsatility and elevated embryonic mortality, alongside a 20–35% reduction in voluntary feed intake. Finishing pigs exhibit severe gut barrier compromise, elevated serum endotoxins, reduced lean meat accretion, and higher carcass drip loss [69,70]. Mineral optimization with supplemental organic zinc (40–60 mg/kg DM) improves intestinal barrier integrity and restores average daily gain by 5–8% in heat-stressed finishing pigs [68].

### 5.3. Rabbit (Lagomorph) Vulnerabilities

Domestic rabbits (Oryctolagus cuniculus) possess dense fur coverage and non-functional sweat glands across the body, with thermolysis restricted to respiratory moisture evaporation and convective vascular exchange across the highly vascularized ear pinnae. Ambient temperatures above 28 °C induce severe hyperthermia, elevating rectal temperatures above 40.5 °C. In breeding does, thermal stress severely suppresses hypothalamic GnRH, reduces conception rates by up to 40–50%, impairs follicular steroidogenesis, and increases post-implantation fetal resorption. In pregnant lagomorphs exposed to severe summer heat stress, dietary supplementation with bioactive polyphenols (curcumin) and vitamin D_3_ significantly ameliorated maternal oxidative stress, restored progesterone synthesis, and improved kit birth weight and postnatal viability [71]. In growing kits, heat stress depresses daily weight gain by 20–30% and increases mortality due to cecal dysbiosis and enterotoxemia.

### 5.4. Poultry Vulnerabilities

**Figure 4:**
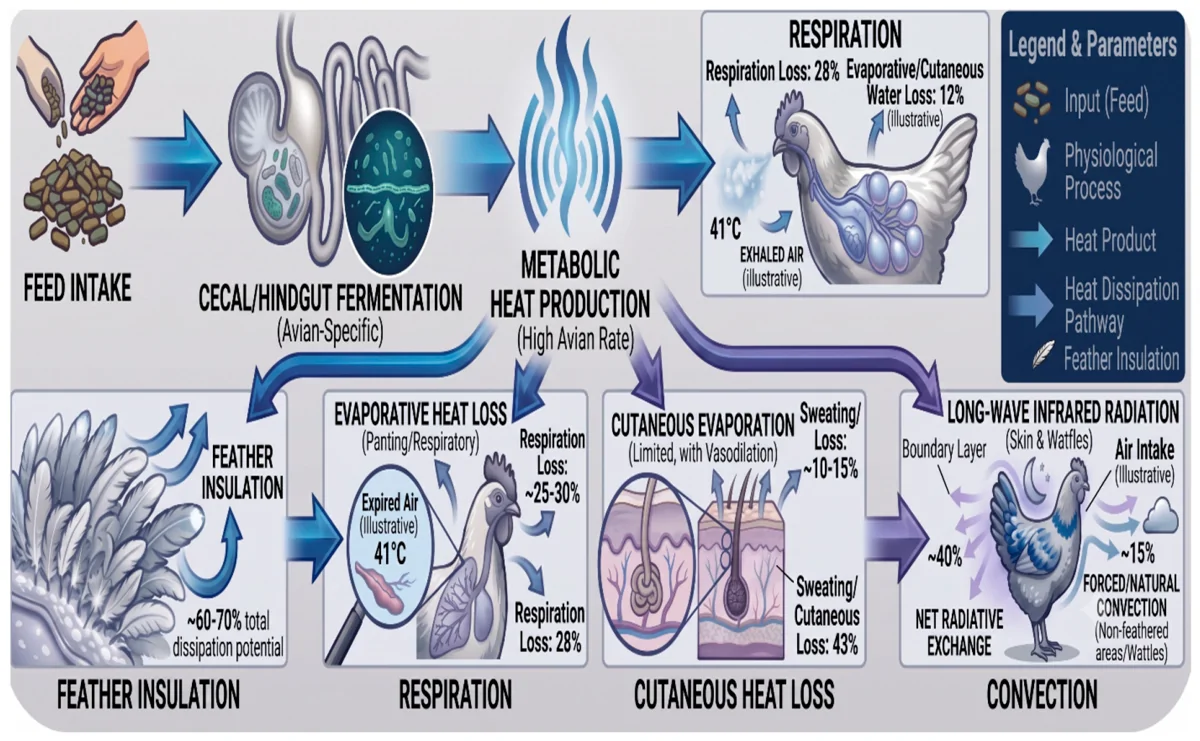
Schematic of heat balance in the chicken. This diagram illustrates the cascading flow from feed intake through avian-specific physiological processes to external dissipation in a chicken model. Key pathways for metabolic heat generation, feather insulation, and specific heat loss routes (respiratory and cutaneous) are defined for physiological analysis.

Poultry are challenged by high metabolic rates and insulative properties of feathers, which combine to limit heat loss. Birds have high core temperatures (~41–42 °C), and the fast metabolism of broilers can easily push animals beyond their thermoneutral zone with small increases in ambient temperature (broiler thermoneutrality often 28–32 °C; adult layers ~18–24 °C) [72]. Feathers impede convective and conductive heat loss, so the main avenues of heat loss are respiratory evaporative cooling (panting) and heat transfer through unfeathered areas (head and shanks); sustained panting can lead to respiratory alkalosis, electrolyte imbalance, and impaired oxygen delivery [73]. Heat stress decreases feed intake, growth, and egg production; broilers under heat stress have reduced lean deposition and increased fat and meat-quality changes (pH shifts, drip loss, and reduced tenderness) associated with altered muscle metabolism, oxidative stress, and upregulation of heat-shock proteins (HSP70/90) [74]. Heat damages egg number, egg mass, shell quality, and internal quality by disturbing calcium and bicarbonate transport in the oviduct. Figure 4 shows a diagram of heat balance in the chicken.

With climate change increasing the frequency and intensity of heat events, intensive poultry systems—with high stocking densities and multi-tier housing—are vulnerable to localized overheating and uneven air distribution [59]. Integrated strategies combining genetic selection for heat tolerance, nutritional adjustments (electrolyte balance, betaine, and antioxidants), housing redesign for better airflow and cooling, and precision monitoring (infrared thermography and vocalization analysis) offer the best path to sustain performance, welfare, and product quality in a warming world [42,75].

## 6. Health and Immune Function

Severe heat stress in livestock leads to profound immunosuppression. This paradox of increased susceptibility to infection and chronic low-grade inflammation further impairs immune competence. This is because of multiple interrelated mechanisms, including decreased numbers of circulating lymphocytes, impaired neutrophil function (chemotaxis, phagocytosis, and antimicrobial peptide synthesis), decreased natural killer cell activity, altered cytokine profiles, and decreased antibody responses to vaccination [66,67]. Especially vulnerable are mucosal compartments such as the gut-associated lymphoid tissue and the mammary gland. The thermal challenge impairs intestinal barrier integrity and induces bacterial translocation and also compromises the local immune responses that restrict pathogens, thus increasing the risk of enteric and systemic infections [65]. For instance, dairy cows under heat stress have decreased neutrophil chemotaxis and antimicrobial peptide synthesis, which increases the risk of bacterial infection under thermal load [76].

Heat stress also impacts adaptive immunity. Impaired T and B cell functions, CD^4+^ helper T cell populations, relative increases in CD^8+^ suppressor/cytotoxic cells, and changes in the CD4/CD8 ratio have been documented, all of which compromise coordinated immune responses [77]. Heat stress inhibits B-cell proliferation and differentiation into antibody-secreting plasma cells with a significant decline in immunoglobulin titers. Therefore, vaccine efficacy is compromised, with studies reporting 30–50% lower post-vaccination antibody titers in heat-stressed animals compared to thermoneutral controls, creating critical protection gaps at a time when exposure risk is heightened [78]. These findings have implications for herd and flock health management and suggest that the timing of vaccination or the immunization strategy during hot periods may be modified or improved.

Targeted nutrition interventions may attenuate heat-induced immunosuppression. Antioxidant and immunomodulatory nutrient benefits have been shown. Vitamin E (1000 to 2000 IU/day) enhances neutrophil function and reduces oxidative damage to lymphocytes. Vitamin C (5–10 g/day) enhances adrenal function and immune cell proliferation. Immune cell glutathione peroxidase activity was maintained at 0.3 mg Se/kg diet. Zinc supplementation at 50–60 mg/kg above basal levels—or formulated to meet target total dietary concentrations within species-specific regulatory safety thresholds—supports immune cell development and functional competence under thermal stress [79], particularly when highly bioavailable organic forms are utilized. Long-chain omega-3 polyunsaturated fatty acids (EPA, DHA) modulate excessive inflammation and pathogen defense. Prebiotics/probiotics maintain gut barrier integrity, and beneficial microbiota can reduce the risk of endotoxemia and systemic infection during heat exposure [80]. Implementation of such nutritional strategies before the onset of heatwaves markedly improves immune resilience and reduces disease incidence under thermal challenge [81].

From a clinical pathology perspective, the epidemiological association of heatwaves with livestock diseases includes severe mammary and respiratory complications. Mastitis incidence has been reported to be 20–40% higher and somatic cell counts 30–50% higher under heat stress from subclinical mammary inflammation caused by dehydration-induced teat sphincter dysfunction, reduced mammary perfusion and immune cell delivery, increased bacterial growth in warm conditions, and reduced pathogen clearance [82,83]. Heat load will dramatically increase respiratory disease that is common in intensive swine and poultry systems with high stocking densities and poor ventilation [84]. Summer heat increases swine pneumonia and respiratory morbidity by 25% to 40%. Poultry are also more susceptible to airsaculitis and respiratory distress during periods of extreme heat, and mortality increases two- to fourfold. Increased panting frequency elevates pathogen exposure within the respiratory tract, thereby heightening susceptibility to infection. Heat stress disrupts pulmonary immunity and mucociliary clearance.

Metabolic disorders are also more likely to occur when there is thermal stress. In dairy cows, heat stress increases the incidence of ketosis (20–30%), fatty liver (15–25%), and displaced abomasum (≈15–20%) due to reduced feed intake and altered metabolic signaling leading to negative energy balance and insulin resistance resulting in fat mobilization and hepatic lipid accumulation [85]. Changes in feeding pattern and rumen motility in heat challenge predispose to ruminal acidosis, with incidence reported to increase 25–35% in hot months. If untreated, severe heat stroke in pigs can be fatal. Mitigation needs to be integrated to treat the combined animal health, welfare and economic burdens of heat-related disease. This includes environmental cooling to reduce thermal load, nutritional support to improve immune and metabolic function, improved biosecurity to minimize exposure to pathogens, and changes in health monitoring and vaccination strategies to address the heat risk [86,87].

## 7. Mitigation and Adaptation Strategies

Evaporative cooling systems (sprinklers and foggers) provide substantial cooling in dry climates [88,89]. Importantly, firing high-pressure misting or fogging systems inside poorly ventilated barns during periods of elevated ambient relative humidity (RH > 60–65%) severely impedes evaporative efficiency, artificially spiking the THI and inadvertently driving animals into lethal heat exhaustion [90,91,92]. In pasture systems, natural or structured shade complements these efforts by blocking 80–90% of solar radiation and lowering body temperature by 1–3 °C at peak heat [15,93]. Combining adequate species-specific shade allowances with rotational grazing enhances feed intake by 15–25% and improves overall animal performance [94]. Consequently, adopting an integrated approach across all heat loss mechanisms yields rapid economic returns, often recovering infrastructure investments within 2–4 years [95,96].

Genomic introgression of the SLICK hair locus (PRLR mutation) into Holstein lines enables cows to sustain 4.0 to 6.0 kg/day greater milk yields during chronic summer thermal stress compared to wild-type herdmates [97,98]. Recent advances in multi-omics frameworks (transcriptomics, proteomics, and metabolomics) have identified key molecular biomarkers of thermal resilience (HSP70, HSP90AA1, SOD2, citrulline, and betaine) [99]. Coupling these multi-omics profiles with real-time Precision Livestock Farming (PLF) IoT streams (computer vision, acoustic vocalization/panting sensors, orbital infrared thermography, and reticulorumen boluses) feeds deep learning architectures (CNN-LSTM neural networks and random forest classifiers) [100,101]. These AI systems predict thermal distress events 24 to 72 h in advance with 88–96% accuracy, autonomously activating variable-speed fans, evaporative pads, and precision nutritional dispensers, saving 20–30% in operational energy [102,103,104]. However, a major socio-technical barrier to large-scale PLF deployment remains the lack of open, standardized API communication protocols among competing equipment manufacturers, creating severe data fragmentation across independent herd management platforms.

### Regional and System-Specific Protocols

- Arid and Semi-Arid Regions (such as Egypt, the Middle East, and Australia): Water-conservation cooling via direct low-pressure cow showers (30–60 s cycles every 5 min at feed bunks) combined with continuous air velocity (≥2.5 m/s); nocturnal feeding regimes (distributing 60–70% of total mixed ration between 20:00 and 04:00); and dietary sodium bicarbonate (1.5–2.0% DM) with potassium chloride (1.0% DM).
- Humid Tropical & Subtropical Systems: Avoid misting when relative humidity exceeds 65%; prioritize high-volume low-speed (HVLS) fans achieving ≥3.0 m/s airspeed at animal level; deploy tropically adapted genetics (SLICK introgression and Girolando crosses); and supplement rumen-protected choline and organic zinc.
- Extensive Pastoral Systems: Agroforestry integration providing ≥4.0 m^2^/head of natural canopy shade; perimeter water troughs within ≤200 m of grazing quadrants; and rotational night-grazing schedules.

## 8. Prospects and Challenges

Sustainable intensification of livestock production under increasing heat stress and increasing productivity while reducing environmental impact and maintaining animal welfare in hot climates is a major challenge for global agriculture [105,106]. This need is especially critical in the tropics, which must continuously manage their heat load with minimal greenhouse gas emissions, water use, and land degradation. Many production systems are already operating close to the thermal limits of animals, and the predicted climate change is likely to increase heat stress beyond the adaptive capacity of existing breeds and management practices, necessitating a fundamental rethinking of livestock systems [86,107]. Integrated approaches to achieve sustainable intensification under heat stress include genetic selection for heat tolerance, improved environmental management, optimized nutrition, and precision livestock technologies. Overall, these elements can enhance productivity with minimal heat losses and environmental impacts [63,108].

Heat is a complex threat and therefore requires an integrated response; there isn’t one size-fits-all solution. Genetic improvement provides long-term cumulative benefits across generations, while environmental and nutritional interventions provide immediate protection during heat events. Precision farming (sensors, automated monitoring, and decision-support systems) provides the opportunity to use resources better, to detect early and to take targeted measures, to reduce costs and to obtain better results [102]. The combined strategies have shown synergistic gains of 20–30% better performance than the single strategies in heat events [86]. But the high capital costs, technical complexity, limited availability of technologies and knowledge in low-income regions, and the need for coordinated action among farmers, researchers, policymakers, and industry stakeholders continue to hamper the adoption of these solutions [105,109].

Assess environmental tradeoffs of strategies for mitigation towards true sustainability. Energy-intensive cooling can add to greenhouse gas emissions when not coupled with renewable energy sources; evaporative cooling can increase water scarcity in arid zones, and increasing reliance on high-quality feeds can result in increased land use and emissions from feed production [110]. Priority interventions should include selective breeding for thermal tolerance, nutritional strategies to enhance feed conversion efficiency, and renewable energy-powered environmental control systems to optimize productivity and reduce ecological impact. Research innovation, enabling policy frameworks and value-chain collaboration, will be needed to develop climate-smart livestock systems that can address heat stress, productivity, and sustainability at the same time [111].

Maternal hyperthermia during critical gestational windows (particularly the dry period in dairy cattle and peri-implantation/late gestation in sows) exerts profound transgenerational epigenetic impacts. Heat-induced placental hypoxia compromises utero-placental blood flow and downregulates glucose (GLUT1/GLUT3) and amino acid (SNAT2) transporters. Epigenetically, thermal stress alters DNA methyltransferase (DNMT1, DNMT3a/b) activities, promoting genome-wide hypo- or hypermethylation in fetal liver, skeletal muscle, and germline cells [112,113]. Furthermore, altered post-translational histone modifications (H3K9me3, H3K27me3) and maternal exosomal microRNAs (miR-21, miR-155, miR-29b) mediate durable metabolic reprogramming. As a consequence, female offspring born to heat-stressed dams exhibit permanent structural hypoplasia of the mammary parenchyma (reduced alveolar epithelial cell proliferation), an innate immune deficit characterized by blunted neutrophil oxidative burst, and an irreversible 10–15% decline in first- and second-lactation milk yields alongside reduced birth weight (5–10%) [114,115].

However, the epigenetic mechanisms of germ cells and their transmission across generations are poorly defined, which prevents targeted mitigation, such as gestational nutrition protection, management of critical windows, or selection for resistance to heat-induced epigenetic reprogramming. Therefore, transgenerational effects can be cumulative, resulting in progressive declines in productivity and welfare under chronic heat, and enhancing the effects of climate change beyond estimates based on direct effects alone [116]. There is very little research on how heat stress interacts with other climate change pressures, such as changes in disease dynamics, changes in the productivity of feed crops, water scarcity and extreme weather, which together determine the resilience of livestock. Social and cultural impacts on smallholder livelihoods, food security and gender roles, as well as economic impacts in developing regions where many livestock and the most vulnerable populations to warming are, are understudied [105]. Closing these gaps and informing proactive, equitable responses to heat stress in livestock systems will require international research coordination, interdisciplinary collaboration across animal science, climate science, economics and social sciences, and increased investment. To address these multifaceted impacts, Table 2 integrates the principal physiological and systemic challenges encountered in heat-stressed livestock with their corresponding multidisciplinary remediation pathways.

## 9. Conclusions

This review synthesized the multidimensional impacts of climate-induced heat stress across farm animals, linking cellular and endocrine dysfunctions to systemic productive and reproductive failures. The physiological cascade—initiated by thermoregulatory strain and shifting from anabolic production to catabolic survival pathways—consistently induces mitochondrial oxidative injury, splanchnic ischemia, and gastrointestinal barrier breakdown (‘leaky gut’) across ruminants, swine, and poultry alike. A critical finding is that vulnerability is highly taxon- and breed-dependent, shaped by metabolic heat of fermentation in cattle and small ruminants, lack of functional sweat glands in swine, and high feather insulation combined with rapid metabolic turnover in poultry.

Single-intervention strategies are inadequate to counter future climate severity. Sustaining livestock resilience demands a coordinated, multi-tier framework: (1) climate-responsive housing combining advanced ventilation and evaporative cooling; (2) species-specific metabolic nutrition incorporating bypass fats, osmolytes, phytogenics, and antioxidant buffers to preserve gut integrity and cellular homeostasis; (3) genomic selection integrating thermotolerance loci (the SLICK locus and HSP variants) with high-producing genetic lines; and (4) real-time precision livestock monitoring for preemptive microclimate management. Finally, successfully safeguarding livestock resilience, animal welfare, and global food systems in an era of accelerating climate change demands rigorous international research coordination that synergistically bridges animal physiology, nutritional biochemistry, genomic breeding, precision engineering, and socio-economic climate policy.

## Figures and Tables

**Table 1:** Illustrates the performance reduction due to heat stress across livestock production traits.

Species/Production Class	Thermal Exposure Index/Temp	Feed Intake Reduction (%)	Primary Productive and Reproductive Impacts	References
Dairy Cattle (*Bos taurus*, Holsteins)	THI ≥ 68–72 Severe: THI ≥ 80	15–35%	Milk yield drops by 10–40%; milk fat and protein drop by 0.2–0.4%; conception rates decline by 30–50%.	[15,37,38]
Beef Cattle (*Bos taurus* vs. *Bos indicus*)	THI ≥ 75–78	10–25%	ADG reduced by 10–20% in *B. taurus*; *B. indicus* (Nellore/Brahman) shows minimal drop until THI > 84.	[39,40]
Growing/Finishing Pigs	28–35 °C (THI ≥ 74)	12–30%	ADG decreases by 15–25%: carcass lean percentage reduced, altered fat composition; sow farrowing rate drops by 10–20%.	[41,42]
Broiler Poultry	32–38 °C	15–30%	Body weight gain decreases by 15–25%; FCR deteriorates by 10–20%; acute mortality increases $\left(2–4\times \right)$.	[23,34]
Laying Poultry	30–36 °C	15–25%	Egg production drops by 10–25%; egg weight and shell thickness decrease by 5–15%; egg breakage rises.	[43,44]
Sheep (Ovine)	30–40 °C THI ≥ 78	10–20%	ADG drops by 10–18%; decreased sperm motility/oocyte viability; wool growth reduced.	[38,45]
Goats (Caprine)	35–42 °C THI ≥ 82	5–15%	Milk yield decreases by 5–15%; higher resistance than sheep; localized indigenous breeds sustain higher intake.	[45,46]

**Table 2:** Integrated Challenges and Multidisciplinary Prospect/Solution Matrix in Heat-Stressed Livestock Systems.

Domain/Challenge	Pathophysiological/Environmental Barrier	Multidisciplinary Prospect/Solution
Digestive heat load and ruminal acidosis	Fermentation heat increment (20–30% of metabolic heat); reduced DMI; ruminal motility stasis.	Rumen-protected bypass fats (0.5–1.0% DM); low-heat synthetic amino acids; high-fiber digestible co-products; DCAD optimization (+38 mEq/100 g).
Gut barrier degradation and dysbiosis	Splanchnic hypoperfusion; tight-junction degradation; LPS systemic endotoxemia and systemic inflammation.	Organic zinc chelates (40–60 mg/kg); selenomethionine (0.3 mg/kg); encapsulation of phytogenics, butyrate, and targeted probiotics/prebiotics.
Transgenerational epigenetic programming	Late-gestation/dry-period maternal hyperthermia altering fetal DNA methylation (DNMTs) and organogenesis.	Maternal dry-period cooling sheds; gestational micronutrient support; early epigenetic biomarker screening in neonatal heifers/gilts.
Cooling infrastructure resource trade-offs	High water usage in evaporative cooling; fossil electricity consumption adding to farm GHG footprint.	Closed-loop heat exchangers; solar/PV-powered variable ventilation; intermittent pulse-soaking logic linked to real-time THI sensors.
PLF deployment and data silos	High initial capital expenditure; proprietary closed sensor APIs; lack of multi-device interoperability.	Open-source ISO-compatible agricultural data standards; edge-computing edge-AI microcontrollers; subsidized regional sensor sharing pools.

## Data Availability

The original contributions presented in this study are included in the article. Further inquiries can be directed to the corresponding authors.
